# Supplementary material for: Promises and Challenges of Predictive Blood Biomarkers for Locally Advanced Rectal Cancer Treated with Neoadjuvant Chemoradiotherapy
Source: Cells. 2023 Jan 26;12(3):413. doi: 10.3390/cells12030413 (PMC9913546; doi:10.3390/cells12030413)
Supplement: Supplementary file 1 [file cells-12-00413-s001.zip › cells-2189517-supplementary.pdf]

Supplementary Material

Table S1. Overview of the studies on predictive value of circulating biomarker in LARC.

Study articles								
authors	study year	number of patients	treatment type	radiation dosage	time to surgery	blood sampling time	measured biomarker	tumor response endpoint
Yang *et al.* [1]	2000-2009	137	5-FU based CRT	45Gy	3.4-12.4 weeks	*pre*-CRT/ *post*-CRT	CEA	pCR
Kim *et al.* [2]	2003-2011	314	5-FU based CRT	45-50Gy	6-8 weeks	*pre*-CRT / *post*-CRT	CEA / CA 19-9 / thrombocytes	TRG (Dworak)
Choi *et al.* [3]	2007-2012	53	5-FU based CRT	45-63Gy	6-8 weeks	pre-CRT	CEA / Hb	pCR
Heo *et al.* [4]	2010-2013	52	5-FU based CRT	44-45Gy	4-8 weeks	*pre*-CRT / during-CRT / *post*-CRT	leukocytes / lymphocytes / Hb / CEA	pCR
Jung *et al.* [5]	2005-2013	984	5-FU based CRT	45-50Gy	6-8 weeks	*pre*-CRT	NLR / PLR / LMR	TRG (Gastrointestinal Pathology Study Group of the Korean Society of Pathologists)
Yang *et al.* [6]	2014-2017	531	n/a	N/A	5-12 weeks	*Pre*-CRT / *post*-CRT	CEA / CA19-9 / Hb	pCR
Cheong *et al.* [7]	2011-2017	145	5-FU based CRT	50.4Gy	6-8 weeks	*Pre*-CRT / *post*-CRT	CEA / NLR / PLR	TRG (AJCC)
Engel *et al.* [8]	2010-2018	331	5-FU or Capecitabine based CRT	45-50.4Gy	1-70 weeks	*pre*-CRT	CEA	pCR
Huang *et al.* [9]	2011-2017	236	5-FU based CRT	45-50.4Gy	n/a	*pre*-CRT /*post*-CRT	CEA / leukocytes / Hb	pCR (ypT0N0)
Gago *et al.* [10]	2012-2017	89	5-FU based CRT	45Gy	n/a	*pre*-CRT	CEA	TRG (Mandard)
Guo *et al.* [11]	2007-2018	751	Capectiabine based CRT	46-50Gy	6-8 weeks	*pre*-CRT	Serum lipids: apoAI, CEA, CA 19-9, TC, TG, HDL-c, LDL-c, apoB	TRG (Mandard)
Zhang *et al.* [12]	2008-2018	432	Capectiabine based CRT	50Gy	6-8 weeks	*pre*-CRT / *post*-CRT	CEA	pCR
Berardi *et al.* [13]	1996-2003	317	5-FU based CRT or RT alone	n/a	4-6 weeks	*pre*-CRT, *post*-CRT *post*-Surgery	Hb	Downstaging
Song *et al.* [14]	2007-2016	674	5-FU or Capecitabine based CRT	45-50.4 Gy	6-8 weeks	pre-CRT / post-CRT	CEA / CA 19-9	Downstaging / pCR
Yeo *et al.* [15]	2009-2013	260	5-FU or Capecitabine based CRT	45 Gy	n/a	*pre*-CRT	CEA / CA 19-9 / Hb	Downstaging / pCR
Restivo *et al.* [16]	1995-2010	260	5-FU or Capecitabine based CRT	45 Gy	minimum 4 weeks	*post*-CRT	CEA	pCR
Jang *et al.* [17]	2004-2009	109	5-FU or Capecitabine based CRT	45 Gy	6-9 weeks	*pre*-CRT / *post*-CRT	CEA / Hb	TRG (Dworak)
Cai *et al.* [18]	2010-2017	284	5-FU or Capecitabine based CRT	46-50.4Gy	n/a	*pre*-CRT/ *post*-CRT	CEA / CA 19-9	TRG (NCCN) / Downstaging
Hu *et al* [19]	2014-2015	149	5-FU or Capecitabine based CRT	46-50.4Gy	n/a	*pre*-CRT/ during-CRT/ *post* CRT	CEA	pCR / TRG
Kitayama *et al.* [20]	2004-2009	73	5-FU based CRT	50.4 Gy	n/a	*Pre*-CRT / *Post*-CRT	CEA / thrombocytes / lymphocytes	pCR
Sawada *et al.* [21]	2005-2014	267	5-FU based CRT	45-50.4 Gy	n/a	*pre*-CRT	CEA / LCR/LMR/NLR/PNI/ CRP to albumin ratio?/ NxCRP/MxCRP/NAR/NxM	TRG (Dworak)

Tawfik *et al.* [22]	2000-2015	98	5-FU based CRT	45-50Gy	n/a	*pre*-CRT /*post*-CRT	LMR/PLR/NLR/NAR / albumin/ CEA / Hb / thrombocytes / WBC	pCR
Krauthamer *et al.* [23]	2002-2007	140	5-FU based CRT	44Gy	6-8 weeks	*pre*-CRT	Albumin /Hb / NLR / Thrombocytes	pCR
Aires *et al.* [24]	2013-2019	171	5-FU or Capecitabine based CRT	45-55Gy	n/a	*pre*-CRT	CRP / CEA / CA 19-9 / Hb / Thrombocytes / NLR	TRG (Ryan)
Dreyer *et al.* [25]	1999-2010	79	5-FU or Capecitabine based CRT	39.6-50Gy	n/a	*pre*-CRT	mGPS / CEA / Hb / NLR / PLR	TRG (Rödel)
Agostini *et al.* [26]	1998-2008	67	5-FU based CRT	50Gy	4-8 weeks	*pre*-CRT / *post*-CRT	cfDNA	TRG (Mandard)
Zitt *et al.* [27]	N/A	26	5-FU based CRT	45Gy	n/a	*Pre*-CRT/ *post*-CRT/ *post*-Surgery	cfDNA	yp Stage
Sun *et al.* [28]	N/A	34	Capecitabine based CRT	50Gy	6-8 weeks	*pre*-CRT / *post*-CRT	cfDNA / KRAS mutation / MGMT promoter metyhlation	TRG (Dworak)
Carpinetti *et al.* [29]	N/A	4	5-FU based CRT	50.4-54Gy	n/a	*pre*-CRT / during-CRT / *post*-CRT	ctDNA	TRG (Dworak)
Sclafani *et al.* [30]	N/A	97	Capecitabine based CRT	50.4Gy	4-6 weeks	*pre*-CRT	ctDNA / KRAS and BRAF mutation	CR (Recist 1.1)
Tie *et al.* [31]	2012-2015	159	5-FU based CRT	n/a	n/a	*pre*-CRT / *post*-CRT / *post*-Surgery	ctDNA	TRG (Dworak)
Shalaby *et al.* [32]	N/A	93	Capecitabine based CRT	50.4Gy	6 weeks	*pre*-CRT	cfDNA, MGMT / ERCC 1 promoter methylation	TRG (Dworak)
Schou *et al.* [33]	2009-2014	123	Capecitabine based CRT	54Gy	6-8 weeks	*pre*-CRT / *post*-CRT	cfDNA	pCR
Appelt *et al.* [34]	2005-2008	146	5-FU based CRT	50.4Gy	8 weeks	*pre*-CRT	methylation of NPY gene in ctDNA	TRG (Mandard)
Khakoo *et al.* [35]	2015-2016	47	Capecitabine based CRT	50.4-54Gy	n/a	*pre*-CRT / during CRT / *post*-CRT	ctDNA	mrTRG (MRI) or TRG (Mandard)
Pazdirek *et al.* [36]	2013-2017	36	Capecitabine based CRT	50.4Gy	8-10 weeks	*pre*-CRT / *post*-CRT	ctDNA	TRG (dworak)
Murahashi *et al.* [37]	2017-2018	85	5FU or Capecitabine based CRT	50.4Gy or 25 Gy	n/a	*pre*-CRT / *post*-CRT / *post*-Surgery	ctDNA / CEA	TNM / TRG (Dworak)
Zhou *et al.* [38]	2017-2019	104	Capecitabine based CRT	45-50Gy	8 weeks	*pre*-CRT / during-CRT / *post*-CRT	ctDNA	TNM / TRG (CAP)
McDuff *et al.* [39]	2014-2018	29	5-FU based CRT	50.4Gy	n/a	*pre*-CRT / *post*-CRT	ctDNA	pCR
Wada *et al.* [40]		106	5-FU or capecitabine based CRT	45-50.4Gy	n/a	*pre*-CRT	miRNA / CEA	TRG (Mandard)
Azizian *et al.* [41]	2011-2012	42	5-FU based CRT	not specified	4 weeks	*pre*-CRT / during-CRT / *post*-CRT	miRNA	Lymph node negativity
D'Angelo *et al.* [42]	2007-2011	34	5-FU or Capecitabine based CRT	at least 45Gy	n/a	*pre*-CRT	miRNA	TRG (Mandard)
Hiyoshi *et al.* [43]	2013-2016	94	n/a	50.4Gy	4-8 weeks	*pre*-CRT	miRNA	TRG (Dworak)
Yu *et al.* [44]	2006-2015	87	n/a	50-50.4Gy	6-8 weeks	*pre*-CRT	miRNA	TRG (Mandard)
Meltzers *et al.* [45]	2013-2015	29	Capecitabine based CRT or SCRT	25 or 50 Gy	n/a	*pre*-CRT	Exosomal miRNA	TRG (CAP) or TRG (Bouzourene)
Baek *et al.* [46]	2012-2015	89	5-FU or Capecitabine based CRT	45-504Gy	6-8 weeks	*pre*-CRT	miRNA	TRG (Rödel)

Dreussi *et al.* [47]	N/A	265	5-FU based CRT	50.4-55Gy	n/a	n/a	SNP related to miRNA	pCR / TRG

Legend : 5-FU : 5-fluorouracil, apoA1 : apolipoprotein A1, apoB : apolipoprotein B, CA 19-9 : carbohydrate antigen 19-9, CEA: carcinoembryonic antigen, cfDNA : cell free DNA, ctDNA : circulating tumor DNA, CRT : chemoradiotherapy, CAP : college of american pathologists, CR : complete response, CRP : c-reactive protein, Hb : hemoglobin, HDL-c : high-density lipoprotein cholesterol, LDL-c : low-density lipoprotein cholesterol, LCR : lymphocyte to CRP ratio, LMR : lymphocyte to monocyte ratio, mrTRG : magnetic resonance tumor regression grade, miRNA : micro RNA, mGPS : modified Glasgow prognostic score, MxCRP : monocyte x CRP product NAR : neutrophil to albumin ratio, NLR : neutrophile to lymphocyte ratio, NxCRP : neutrophil x CRP product, NxM : neutrophile x monocyte product, n/a : not available, pCR : pathological complete response, PLR: latelet to lymphocyte ratio, SNP : single nucleotide polymorphism, TG : triglyceride, TC : total cholesterol, TRG: tumor regression grade.

Table S2. Studies exploring CA19-9 as a biomarker predictive of tumor regression following CRT.

Study	Cut-off	N	Measured outcome	Sn	Sp	VPP	VPN	p.value (Univariate)
pre-CRT								
Aires *et al.* [24]	3.5 U/ml	171	TRG 0-1 (Rayan)	39.6%	72.5%	n/a	n/a	ns
Yeo *et al.* [15]	9 U/ml<br>+	260	ypStage 0-1	70.2%	50%	44.3%	74.8%	<0.01(*)
	10 U/ml	260	ypStage 0-1	74.5%	48.2%	44.9%	76.9%	<0.01
Song *et al.* [14]	12.6 U/ml	674	Downstaging	62%	54.5%	52.9%	63.4%	0.0001
Guo *et al.* [11]	35 U/ml	751	TRG 1-2 (Mandard)	n/a	n/a	n/a	n/a	ns
Kim *et al.* [2]	37 U/ml	314	pCR	86.5%	21.5%	13.7%	91.7%	ns
Yang *et al.* [6]	37 U/ml	531	pCR	85%	24.8%	20.8%	87.7%	ns
Cai *et al.* [18]	37 U/ml	284	TRG 0-1 (NCCN)	93.6%	97.7%	39.6%	70.8%	ns
post-CRT & *pre*-surgery								
Song *et al.* [14]	11.3 U/ml	674	Downstaging	58%	49.1%	48.5%	58.6%	ns
pre- and *post* CRT ratio								
Song *et al.* [14]	0.92<br>+	674	pCR	44.9%	66.7%	25.4%	82.7%	0.012
	1.28	674	Downstaging	73.1%	41.7%	50.9%	65.3%	<0.0001(*)
reduction ratio								
Yang *et al.* [6]	n/a	531	pCR	n/a	n/a	n/a	n/a	ns

(*) : also significant in multivariate analysis. Legend : CRT : chemoradiotherapy, NCCN : national comprehensive cancer network, NPV : negative predictive value, ns : non-significant, n/a : not available, N : number of patients, pCR : pathological complete response, yp Stage : pathological stage after neo-adjuvant treatment, PPV : positive predictive value, Sn : sensitivity, Sp : specificity, TRG : tumor regression grade.

Table S3. Studies exploring hemoglobin level as a biomarker predictive of tumor regression following CRT.

Study	Cut-off	N	Measure outcome	Sn	Sp	VPP	VPN	p. value (Univariate)
				pre-CRT				
Yang J. *et al.* [6]	9 g/dl	531	pCR	97%	5.6%	19.2%	88.9%	ns
Choi *et al.* [3]	10 g/dl	53	pCR	90.9%	14.3%	21.7%	85.7%	ns
Krauthamer *et al.* [23]	12 g/dl	140	pCR	63.6%	51%	36.8%	75.8%	ns
Berardi *et al.* [13]	12 g/dl	317	Downstaging	n/a	n/a	n/a	n/a	0.05
Aires *et al.* [24]	12.2g/dl	171	TRG 0-1 (Rayan)	81.4%	39.1%	n/a	n/a	0.0036
Yeo *et al.* [15]	12.5 g/dl	260	Yp Stage 0-1	59.8%	48.2%	39%	68.4%	ns
Heo *et al.* [4]	13.2 g/dl	52	pCR	35.7%	40.5%	18.5%	62.5%	ns
Jang *et al.* [17]	13.5 g/dl	109	TRG 3-4 (Dworak)	42.2%	54.7%	39.6%	57.4%	ns
Tawfik *et al.* [22]	n/a	98	pCR	n/a	n/a	n/a	n/a	0.05
Dreyer *et al.* [25]	13 g/dl (M)<br>11.5 g/dl (F)	79	TRG 3-4 (Rödel)	64.7%	46.5%	48.9%	62.5%	ns
				during CRT				
Huang *et al.* [9]	10 g/dl	236	pCR	83.9%	32.8%	28%	86.8%	0.0159(*)
				post-CRT & *pre*-surgery				
Tawfik *et al.* [22]	n/a	98	pCR	n/a	n/a	n/a	n/a	ns

(*) : also significant in multivariate analysis. Legend : CRT : chemoradiotherapy, NPV : negative predictive value, ns : non-significant, n/a : not available, N : number of patients, pCR : pathological complete response, yp Stage : pathological stage after neo-adjuvant treatment, PPV : positive predictive value, Sn : sensitivity, Sp : specificity, TRG : tumor regression grade.

Table S4. Studies exploring albumin or CRP levels as biomarkers predictive of tumor regression following CRT.

Study	Cut-off	N	Measure outcome	Sn	Sp	VPP	VPN	p value (univariate)
				albumin				
				pre-CRT				
Krauthamer *et al.* [23]	3.5 mg/dl	140	pCR	86.4%	24.5%	33.9%	80%	ns
Tawfik *et al.* [22]	n/a	98	pCR	n/a	n/a	n/a	n/a	ns
				Post-CRT				

Tawfik *et al.* [22]	n/a	98	pCR	n/a	n/a	n/a	n/a	ns
				CRP				
				pre-CRT				
Aires *et al.* [24]	3.5 mg/L	171	TRG 0-1 (Rayan)	54.8%	93.5%	n/a	n/a	<0.0001(*)
				mGPS				
Dreyer *et al.* [25]	mGPS 0 (vs mGPS 1-2)	79	TRG 3-4 (Rödel)	88%	44.7%	51.1%	85%	0.022(*)

(*) : also significant in multivariate analysis. Legend : CRT : chemoradiotherapy, NPV : negative predictive value, ns : non-significant, n/a : not available, N : number of patients, pCR : pathological complete response, PPV : positive predictive value, Sn : sensitivity, Sp : specificity, TRG : tumor regression grade.

Table S5. Studies exploring leukocyte and lymphocyte counts as well as NLR, NAR, LMR and PLR ratios as biomarkers predictive of tumor regression following CRT.

Study	Cut-off	N	Measure outcome	Sn	Sp	VPP	VPN	p value (univariate)
				leukocyte Count				
				pre-CRT				
Heo *et al.* [4]	7.3 G/l	52	pCR	83.3%	45.7%	34.5%	88.9%	ns
Tawfik *et al.* [22]	n/a	98	pCR	n/a	n/a	n/a	n/a	ns
				post-CRT *pre*-surgery				
Tawfik *et al.* [22]	n/a	98	pCR	n/a	n/a	n/a	n/a	ns
				during CRT				
Huang *et al.* [9]	3 G/l	236	pCR	51.8%	36.3%	20.3%	70.1%	ns
				lymphocytes				
				pre-CRT				
Kitayama *et al.* [20]	n/a	73	pCR	n/a	n/a	n/a	n/a	0.02(*)
Heo *et al.* [4]	1.996 G/l	52	pCR	42.9%	47.4%	23.1%	75%	ns
				during treatment (*post*-4 weeks)				
Heo *et al.* [4]	X	52	pCR	42.9%	47.4%	23.1%	75%	ns
				post-4 weeks lymphocytes/*pre*-CRT lymphocytes ratio				
Heo *et al.* [4]	0.35	52	pCR	85.7%	57.9%	42.9%	91.7%	0.01(*)
				during treatment (*post*-8 weeks)				
Heo *et al.* [4]	X	52	pCR	22.2%	64.3%	16.7%	72%	ns

post-8 weeks lymphocytes/*pre*-CRT lymphocytes ratio								
Heo *et al.* [4]	0.47	52	pCR	33.3%	53.6%	18.8%	71.4%	ns
during treatment (*post*-12 weeks)								
Heo *et al.* [4]	1.033 G/l	52	pCR	66.7%	26.1%	26.1%	66.7%	ns
post-12 weeks lymphocytes/*pre*-CRT lymphocytes ratio								
Heo *et al.* [4]	0.53	52	pCR	55.6%	47.8%	29.4%	73.3%	ns
neutrophil to lymphocyte ratio (NLR)								
pre-CRT								
Jung *et al.* [5]	1.7	984	TRG (total-near total)	47.2%	57.5%	44.7%	59.9%	ns
Aires *et al.* [24]	2.3	171	TRG 0-1 (Rayan)	51%	58%	n/a	n/a	ns
Cheong *et al.* [7]	2.8	145	pCR	n/a	n/a	n/a	n/a	0.013(*)
Krauthamer *et al.* [23]	5	140	pCR	77.3%	40.8%	37%	80%	ns
Dreyer *et al.* [25]	5	79	TRG 3-4 (Rödel)	91.4%	25%	49.2%	78.6%	ns
Sawada *et al.* [21]	n/a	267	TRG 3-4 (Dworak)	n/a	n/a	n/a	n/a	ns
Tawfik *et al.* [22]	n/a	98	pCR	n/a	n/a	n/a	n/a	0.012
post-CRT								
Tawfik *et al.* [22]	n/a	98	pCR	n/a	n/a	n/a	n/a	ns
neutrophil to albumin ratio (NAR)								
pre-CRT								
Sawada *et al.*[21]	n/a	267	TRG 3-4 (Dworak)	n/a	n/a	n/a	n/a	ns
Tawfik *et al.* [22]	n/a	98	pCR	n/a	n/a	n/a	n/a	0.002(*)
post-CRT								
Tawfik *et al.* [22]	n/a	98	pCR	n/a	n/a	n/a	n/a	0.05
lymphocyte to monocyte ratio (LMR)								
pre-CRT								
Sawada *et al.* [21]	n/a	267	TRG 3-4 (Dworak)	n/a	n/a	n/a	n/a	ns
Tawfik *et al.* [22]	n/a	98	pCR	n/a	n/a	n/a	n/a	ns
Jung *et al.* [5]	6.8	984	TRG (total-near total)	12.2%	86.3%	44.7%	58.2%	ns
post-CRT *pre*-Surgery								

Tawfik *et al.* [22]	n/a	98	pCR	n/a	n/a	n/a	n/a	ns
			platelet to lymphocyte ratio (PLR)					
			pre-CRT					
Jung *et al.* [5]	92.88	984	TRG (total-near total)	20.2%	75.7%	37.8%	56.6%	ns
Cheong *et al.* [7]	138.2	145	pCR	n/a	n/a	n/a	n/a	n/a
Dreyer *et al.* [25]	300	79	TRG 3-4 (Rödel)	88.6%	27.3%	49.2%	75%	ns
Tawfik *et al.* [22]	n/a	98	pCR	n/a	n/a	n/a	n/a	ns
			post-CRT					
Tawfik *et al.* [22]	n/a	98	pCR	n/a	n/a	n/a	n/a	ns
			neutrophil and monocyte product (N x M) *pre*-CRT					
Sawada *et al.* [21]	1,440,000	267	TRG 3-4 (Dworak)	n/a	n/a	n/a	n/a	0.001(*)
			lymphocyte to CRP ratio (LCR) (*pre*-CRT)					
Sawada *et al.* [21]	18.141	267	TRG 3-4 (Dworak)	n/a	n/a	n/a	n/a	0.007(*)

(*) : also significant in multivariate analysis. Legend : CRT : chemoradiotherapy, NPV : negative predictive value, ns : non-significant, n/a : not available, N : number of patients, pCR : pathological complete response, PPV : positive predictive value, Sn : sensitivity, Sp : specificity, TRG : tumor regression grade.

1. Yang, K.L.; Yang, S.H.; Liang, W.Y.; Kuo, Y.J.; Lin, J.K.; Lin, T.C.; Chen, W.S.; Jiang, J.K.; Wang, H.S.; Chang, S.C.; et al. Carcinoembryonic antigen (CEA) level, CEA ratio, and treatment outcome of rectal cancer patients receiving pre-operative chemoradiation and surgery. *Radiat Oncol* **2013**, *8*, 43, doi:10.1186/1748-717X-8-43.
2. Kim, H.J.; Choi, G.S.; Park, J.S.; Park, S.; Kawai, K.; Watanabe, T. Clinical significance of thrombocytosis before preoperative chemoradiotherapy in rectal cancer: predicting pathologic tumor response and oncologic outcome. *Ann Surg Oncol* **2015**, *22*, 513-519, doi:10.1245/s10434-014-3988-8.
3. Choi, E.; Kim, J.H.; Kim, O.B.; Kim, M.Y.; Oh, Y.K.; Baek, S.G. Predictors of pathologic complete response after preoperative concurrent chemoradiotherapy of rectal cancer: a single center experience. *Radiat Oncol J* **2016**, *34*, 106-112, doi:10.3857/roj.2015.01585.
4. Heo, J.; Chun, M.; Noh, O.K.; Oh, Y.T.; Suh, K.W.; Park, J.E.; Cho, O. Sustaining Blood Lymphocyte Count during Preoperative Chemoradiotherapy as a Predictive Marker for Pathologic Complete Response in Locally Advanced Rectal Cancer. *Cancer Res Treat* **2016**, *48*, 232-239, doi:10.4143/crt.2014.351.
5. Jung, S.W.; Park, I.J.; Oh, S.H.; Yeom, S.S.; Lee, J.L.; Yoon, Y.S.; Kim, C.W.; Lim, S.B.; Lee, J.B.; Yu, C.S.; et al. Association of immunologic markers from complete blood counts with the response to preoperative chemoradiotherapy and prognosis in locally advanced rectal cancer. *Oncotarget* **2017**, *8*, 59757-59765, doi:10.18632/oncotarget.15760.
6. Yang, J.; Ling, X.; Tang, W.; Hu, D.; Zhou, H.; Yin, G. Analyses of predictive factors for pathological complete remission in neoadjuvant therapy for locally advanced rectal cancer. *J BUON* **2019**, *24*, 77-83.
7. Cheong, C.; Shin, J.S.; Suh, K.W. Prognostic value of changes in serum carcinoembryonic antigen levels for preoperative chemoradiotherapy response in locally advanced rectal cancer. *World J Gastroenterol* **2020**, *26*, 7022-7035, doi:10.3748/wjg.v26.i44.7022.
8. Engel, R.M.; Oliva, K.; Koulis, C.; Yap, R.; McMurrick, P.J. Predictive factors of complete pathological response in patients with locally advanced rectal cancer. *Int J Colorectal Dis* **2020**, *35*, 1759-1767, doi:10.1007/s00384-020-03633-8.
9. Huang, C.M.; Huang, C.W.; Ma, C.J.; Yeh, Y.S.; Su, W.C.; Chang, T.K.; Tsai, H.L.; Juo, S.H.; Huang, M.Y.; Wang, J.Y. Predictive Value of FOLFOX-Based Regimen, Long Interval, Hemoglobin Levels and Clinical Negative Nodal Status, and Postchemoradiotherapy CEA Levels for Pathological Complete Response in Patients with Locally Advanced Rectal Cancer after Neoadjuvant Chemoradiotherapy. *J Oncol* **2020**, *2020*, 9437684, doi:10.1155/2020/9437684.
10. Gago, T.; Caldeira, P.; Cunha, A.C.; Campelo, P.; Guerreiro, H. Can we optimize CEA as a response marker in rectal cancer? *Rev Esp Enferm Dig* **2021**, *113*, 423-428, doi:10.17235/reed.2020.7321/2020.
11. Guo, S.P.; Chen, C.; Zeng, Z.F.; Wang, Q.X.; Jiang, W.; Gao, Y.H.; Chang, H. Serum Apolipoprotein A-I Predicts Response of Rectal Cancer to Neoadjuvant Chemoradiotherapy. *Cancer Manag Res* **2021**, *13*, 2623-2631, doi:10.2147/CMAR.S302677.
12. Zhang, Q.; Liang, J.; Chen, J.; Mei, S.; Wang, Z. Predictive Factors for Pathologic Complete Response Following Neoadjuvant Chemoradiotherapy for Rectal Cancer. *Asian Pac J Cancer Prev* **2021**, *22*, 1607-1611, doi:10.31557/APJCP.2021.22.5.1607.
13. Berardi, R.; Braconi, C.; Mantello, G.; Scartozzi, M.; Del Prete, S.; Luppi, G.; Martinelli, R.; Fumagalli, M.; Valeri, G.; Bearzi, I.; et al. Anemia may influence the outcome of patients undergoing neo-adjuvant treatment of rectal cancer. *Ann Oncol* **2006**, *17*, 1661-1664, doi:10.1093/annonc/mdl285.
14. Song, J.; Huang, X.; Chen, Z.; Chen, M.; Lin, Q.; Li, A.; Chen, Y.; Xu, B. Predictive value of carcinoembryonic antigen and carbohydrate antigen 19-9 related to downstaging to stage 0-I after neoadjuvant chemoradiotherapy in locally advanced rectal cancer. *Cancer Manag Res* **2018**, *10*, 3101-3108, doi:10.2147/CMAR.S166417.
15. Yeo, S.G.; Kim, D.Y.; Kim, T.H.; Kim, S.Y.; Baek, J.Y.; Chang, H.J.; Park, J.W.; Oh, J.H. Carbohydrate antigen 19-9 levels associated with pathological responses to preoperative chemoradiotherapy in rectal cancer. *Asian Pac J Cancer Prev* **2014**, *15*, 5383-5387, doi:10.7314/apjcp.2014.15.13.5383.
16. Restivo, A.; Zorcolo, L.; Cocco, I.M.; Manunza, R.; Margiani, C.; Marongiu, L.; Casula, G. Elevated CEA levels and low distance of the tumor from the anal verge are predictors of incomplete response to chemoradiation in patients with rectal cancer. *Ann Surg Oncol* **2013**, *20*, 864-871, doi:10.1245/s10434-012-2669-8.
17. Jang, N.Y.; Kang, S.B.; Kim, D.W.; Kim, J.H.; Lee, K.W.; Kim, I.A.; Kim, J.S. The role of carcinoembryonic antigen after neoadjuvant chemoradiotherapy in patients with rectal cancer. *Dis Colon Rectum* **2011**, *54*, 245-252, doi:10.1007/DCR.0b013e3181fcee68.
18. Cai, Z.; Huang, L.; Chen, Y.; Xie, X.; Zou, Y.; Lan, P.; Wu, X. CEA Decline Predicts Tumor Regression and Prognosis in Locally Advanced Rectal Cancer Patients with Elevated Baseline CEA. *J Cancer* **2020**, *11*, 6565-6570, doi:10.7150/jca.49252.
19. Hu, H.; Huang, J.; Lan, P.; Wang, L.; Huang, M.; Wang, J.; Deng, Y. CEA clearance pattern as a predictor of tumor response to neoadjuvant treatment in rectal cancer: a post-hoc analysis of FOWARC trial. *BMC Cancer* **2018**, *18*, 1145, doi:10.1186/s12885-018-4997-y.
20. Kitayama, J.; Yasuda, K.; Kawai, K.; Sunami, E.; Nagawa, H. Circulating lymphocyte number has a positive association with tumor response in neoadjuvant chemoradiotherapy for advanced rectal cancer. *Radiat Oncol* **2010**, *5*, 47, doi:10.1186/1748-717X-5-47.
21. Sawada, R.; Akiyoshi, T.; Kitagawa, Y.; Hiyoshi, Y.; Mukai, T.; Nagasaki, T.; Yamaguchi, T.; Konishi, T.; Yamamoto, N.; Ueno, M.; et al. Systemic Inflammatory Markers Combined with Tumor-Infiltrating Lymphocyte Density for the Improved Prediction of Response to Neoadjuvant Chemoradiotherapy in Rectal Cancer. *Ann Surg Oncol* **2021**, *28*, 6189-6198, doi:10.1245/s10434-021-09975-z.

22. Tawfik, B.; Mokdad, A.A.; Patel, P.M.; Li, H.C.; Huerta, S. The neutrophil to albumin ratio as a predictor of pathological complete response in rectal cancer patients following neoadjuvant chemoradiation. *Anticancer Drugs* **2016**, *27*, 879-883, doi:10.1097/CAD.0000000000000411.
23. Krauthamer, M.; Rouvinov, K.; Ariad, S.; Man, S.; Walfish, S.; Pinsk, I.; Sztarker, I.; Charkovsky, T.; Lavrenkov, K. A study of inflammation-based predictors of tumor response to neoadjuvant chemoradiotherapy for locally advanced rectal cancer. *Oncology* **2013**, *85*, 27-32, doi:10.1159/000348385.
24. Aires, F.; Rodrigues, D.; Lamas, M.P.; Herdeiro, M.T.; Figueiras, A.; Oliveira, M.J.; Marques, M.; Pinto, A.T. C-Reactive Protein as Predictive Biomarker for Response to Chemoradiotherapy in Patients with Locally Advanced Rectal Cancer: A Retrospective Study. *Cancers (Basel)* **2022**, *14*, doi:10.3390/cancers14030491.
25. Dreyer, S.B.; Powell, A.G.; McSorley, S.T.; Waterston, A.; Going, J.J.; Edwards, J.; McMillan, D.C.; Horgan, P.G. The Pretreatment Systemic Inflammatory Response is an Important Determinant of Poor Pathologic Response for Patients Undergoing Neoadjuvant Therapy for Rectal Cancer. *Ann Surg Oncol* **2017**, *24*, 1295-1303, doi:10.1245/s10434-016-5684-3.
26. Agostini, M.; Pucciarelli, S.; Enzo, M.V.; Del Bianco, P.; Briarava, M.; Bedin, C.; Maretto, I.; Friso, M.L.; Lonardi, S.; Mescoli, C.; et al. Circulating cell-free DNA: a promising marker of pathologic tumor response in rectal cancer patients receiving preoperative chemoradiotherapy. *Ann Surg Oncol* **2011**, *18*, 2461-2468, doi:10.1245/s10434-011-1638-y.
27. Zitt, M.; Muller, H.M.; Rochel, M.; Schwendinger, V.; Zitt, M.; Goebel, G.; Devries, A.; Margreiter, R.; Oberwalder, M.; Zeillinger, R.; et al. Circulating cell-free DNA in plasma of locally advanced rectal cancer patients undergoing preoperative chemoradiation: a potential diagnostic tool for therapy monitoring. *Dis Markers* **2008**, *25*, 159-165, doi:10.1155/2008/598071.
28. Sun, W.; Sun, Y.; Zhu, M.; Wang, Z.; Zhang, H.; Xin, Y.; Jiang, G.; Guo, X.; Zhang, Z.; Liu, Y. The role of plasma cell-free DNA detection in predicting preoperative chemoradiotherapy response in rectal cancer patients. *Oncol Rep* **2014**, *31*, 1466-1472, doi:10.3892/or.2013.2949.
29. Carpinetti, P.; Donnard, E.; Bettoni, F.; Asprino, P.; Koyama, F.; Rozanski, A.; Sabbaga, J.; Habr-Gama, A.; Parmigiani, R.B.; Galante, P.A.; et al. The use of personalized biomarkers and liquid biopsies to monitor treatment response and disease recurrence in locally advanced rectal cancer after neoadjuvant chemoradiation. *Oncotarget* **2015**, *6*, 38360-38371, doi:10.18632/oncotarget.5256.
30. Sclafani, F.; Chau, I.; Cunningham, D.; Hahne, J.C.; Vlachogiannis, G.; Eltahir, Z.; Lampis, A.; Braconi, C.; Kalaitzaki, E.; De Castro, D.G.; et al. KRAS and BRAF mutations in circulating tumour DNA from locally advanced rectal cancer. *Sci Rep* **2018**, *8*, 1445, doi:10.1038/s41598-018-19212-5.
31. Tie, J.; Cohen, J.D.; Wang, Y.; Li, L.; Christie, M.; Simons, K.; Elsaleh, H.; Kosmider, S.; Wong, R.; Yip, D.; et al. Serial circulating tumour DNA analysis during multimodality treatment of locally advanced rectal cancer: a prospective biomarker study. *Gut* **2019**, *68*, 663-671, doi:10.1136/gutjnl-2017-315852.
32. Shalaby, S.M.; El-Shal, A.S.; Abdelaziz, L.A.; Abd-Elbary, E.; Khairy, M.M. Promoter methylation and expression of DNA repair genes MGMT and ERCC1 in tissue and blood of rectal cancer patients. *Gene* **2018**, *644*, 66-73, doi:10.1016/j.gene.2017.10.056.
33. Schou, J.V.; Larsen, F.O.; Sorensen, B.S.; Abrantes, R.; Boysen, A.K.; Johansen, J.S.; Jensen, B.V.; Nielsen, D.L.; Spindler, K.L. Circulating cell-free DNA as predictor of treatment failure after neoadjuvant chemo-radiotherapy before surgery in patients with locally advanced rectal cancer. *Ann Oncol* **2018**, *29*, 610-615, doi:10.1093/annonc/mdx778.
34. Appelt, A.L.; Andersen, R.F.; Lindebjerg, J.; Jakobsen, A. Prognostic Value of Serum NPY Hypermethylation in Neoadjuvant Chemoradiotherapy for Rectal Cancer: Secondary Analysis of a Randomized Trial. *Am J Clin Oncol* **2020**, *43*, 9-13, doi:10.1097/COC.0000000000000609.
35. Khakoo, S.; Carter, P.D.; Brown, G.; Valeri, N.; Picchia, S.; Bali, M.A.; Shaikh, R.; Jones, T.; Begum, R.; Rana, I.; et al. MRI Tumor Regression Grade and Circulating Tumor DNA as Complementary Tools to Assess Response and Guide Therapy Adaptation in Rectal Cancer. *Clin Cancer Res* **2020**, *26*, 183-192, doi:10.1158/1078-0432.CCR-19-1996.
36. Pazdirek, F.; Minarik, M.; Benesova, L.; Halkova, T.; Belsanova, B.; Macek, M.; Stepanek, L.; Hoch, J. Monitoring of Early Changes of Circulating Tumor DNA in the Plasma of Rectal Cancer Patients Receiving Neoadjuvant Concomitant Chemoradiotherapy: Evaluation for Prognosis and Prediction of Therapeutic Response. *Front Oncol* **2020**, *10*, 1028, doi:10.3389/fonc.2020.01028.
37. Murahashi, S.; Akiyoshi, T.; Sano, T.; Fukunaga, Y.; Noda, T.; Ueno, M.; Zembutsu, H. Serial circulating tumour DNA analysis for locally advanced rectal cancer treated with preoperative therapy: prediction of pathological response and postoperative recurrence. *Br J Cancer* **2020**, *123*, 803-810, doi:10.1038/s41416-020-0941-4.
38. Zhou, J.; Wang, C.; Lin, G.; Xiao, Y.; Jia, W.; Xiao, G.; Liu, Q.; Wu, B.; Wu, A.; Qiu, H.; et al. Serial Circulating Tumor DNA in Predicting and Monitoring the Effect of Neoadjuvant Chemoradiotherapy in Patients with Rectal Cancer: A Prospective Multicenter Study. *Clin Cancer Res* **2021**, *27*, 301-310, doi:10.1158/1078-0432.CCR-20-2299.
39. McDuff, S.G.R.; Hardiman, K.M.; Ulintz, P.J.; Parikh, A.R.; Zheng, H.; Kim, D.W.; Lennerz, J.K.; Hazar-Rethinam, M.; Van Seventer, E.E.; Fetter, I.J.; et al. Circulating Tumor DNA Predicts Pathologic and Clinical Outcomes Following Neoadjuvant Chemoradiation and Surgery for Patients With Locally Advanced Rectal Cancer. *JCO Precis Oncol* **2021**, *5*, doi:10.1200/PO.20.00220.
40. Wada, Y.; Shimada, M.; Morine, Y.; Ikemoto, T.; Saito, Y.; Zhu, Z.; Wang, X.; Etxart, A.; Park, Y.; Bujanda, L.; et al. Circulating miRNA Signature Predicts Response to Preoperative Chemoradiotherapy in Locally Advanced Rectal Cancer. *JCO Precis Oncol* **2021**, *5*, doi:10.1200/PO.21.00015.

41. Azizian, A.; Kramer, F.; Jo, P.; Wolff, H.A.; Beissbarth, T.; Skarupke, R.; Bernhardt, M.; Grade, M.; Ghadimi, B.M.; Gaedcke, J. Preoperative Prediction of Lymph Node Status by Circulating Mir-18b and Mir-20a During Chemoradiotherapy in Patients with Rectal Cancer. *World J Surg* **2015**, *39*, 2329-2335, doi:10.1007/s00268-015-3083-8.
42. D'Angelo, E.; Fassan, M.; Maretto, I.; Pucciarelli, S.; Zanon, C.; Digito, M.; Rugge, M.; Nitti, D.; Agostini, M. Serum miR-125b is a non-invasive predictive biomarker of the pre-operative chemoradiotherapy responsiveness in patients with rectal adenocarcinoma. *Oncotarget* **2016**, *7*, 28647-28657, doi:10.18632/oncotarget.8725.
43. Hiyoshi, Y.; Akiyoshi, T.; Inoue, R.; Murofushi, K.; Yamamoto, N.; Fukunaga, Y.; Ueno, M.; Baba, H.; Mori, S.; Yamaguchi, T. Serum miR-143 levels predict the pathological response to neoadjuvant chemoradiotherapy in patients with locally advanced rectal cancer. *Oncotarget* **2017**, *8*, 79201-79211, doi:10.18632/oncotarget.16760.
44. Yu, J.; Li, N.; Wang, X.; Ren, H.; Wang, W.; Wang, S.; Song, Y.; Liu, Y.; Li, Y.; Zhou, X.; et al. Circulating serum microRNA-345 correlates with unfavorable pathological response to preoperative chemoradiotherapy in locally advanced rectal cancer. *Oncotarget* **2016**, *7*, 64233-64243, doi:10.18632/oncotarget.11649.
45. Meltzer, S.; Bjornetro, T.; Lyckander, L.G.; Flatmark, K.; Dueland, S.; Samiappan, R.; Johansen, C.; Kalanxhi, E.; Ree, A.H.; Redalen, K.R. Circulating Exosomal miR-141-3p and miR-375 in Metastatic Progression of Rectal Cancer. *Transl Oncol* **2019**, *12*, 1038-1044, doi:10.1016/j.tranon.2019.04.014.
46. Baek, D.W.; Kim, G.; Kang, B.W.; Kim, H.J.; Park, S.Y.; Park, J.S.; Choi, G.S.; Kang, M.K.; Hur, K.; Kim, J.G. High expression of microRNA-199a-5p is associated with superior clinical outcomes in patients with locally advanced rectal cancer. *J Cancer Res Clin Oncol* **2020**, *146*, 105-115, doi:10.1007/s00432-019-03099-4.
47. Dreussi, E.; Pucciarelli, S.; De Paoli, A.; Polesel, J.; Canzonieri, V.; Agostini, M.; Friso, M.L.; Belluco, C.; Buonadonna, A.; Lonardi, S.; et al. Predictive role of microRNA-related genetic polymorphisms in the pathological complete response to neoadjuvant chemoradiotherapy in locally advanced rectal cancer patients. *Oncotarget* **2016**, *7*, 19781-19793, doi:10.18632/oncotarget.7757.
